# Ribonucleoprotein Granules: Between Stress and Transposable Elements

**DOI:** 10.3390/biom13071027

**Published:** 2023-06-23

**Authors:** Sungjin Moon, Sim Namkoong

**Affiliations:** 1 Department of Biological Sciences, Kangwon National University, Chuncheon 24341, Republic of Korea; 2 Department of Biochemistry, Kangwon National University, Chuncheon 24341, Republic of Korea

**Keywords:** transposable element, retrotransposon, RNP granules, stress granule, P-body, post-transcriptional regulation

## Abstract

Transposable elements (TEs) are DNA sequences that can transpose and replicate within the genome, leading to genetic changes that affect various aspects of host biology. Evolutionarily, hosts have also developed molecular mechanisms to suppress TEs at the transcriptional and post-transcriptional levels. Recent studies suggest that stress-induced formation of ribonucleoprotein (RNP) granules, including stress granule (SG) and processing body (P-body), can play a role in the sequestration of TEs to prevent transposition, suggesting an additional layer of the regulatory mechanism for TEs. RNP granules have been shown to contain factors involved in RNA regulation, including mRNA decay enzymes, RNA-binding proteins, and noncoding RNAs, which could potentially contribute to the regulation of TEs. Therefore, understanding the interplay between TEs and RNP granules is crucial for elucidating the mechanisms for maintaining genomic stability and controlling gene expression. In this review, we provide a brief overview of the current knowledge regarding the interplay between TEs and RNP granules, proposing RNP granules as a novel layer of the regulatory mechanism for TEs during stress.

## 1. Invasion of Transposable Elements (TEs) and Host Defense System

**Figure 1:**
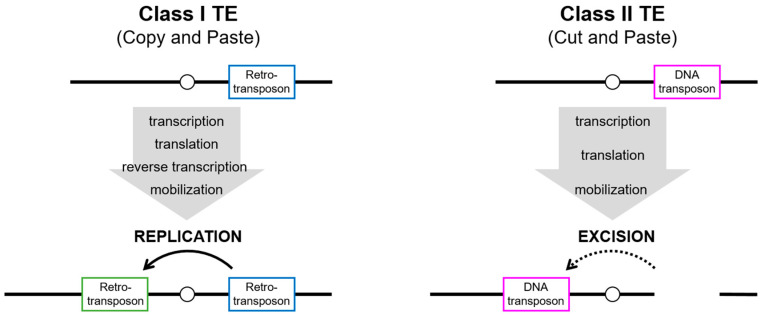
Mechanisms of transposition of class I and class II TEs. Class I TEs (retrotransposons) replicate the element through transcription and translation of the enzymatic machinery, followed by reverse transcription. The new copy (green box) is inserted elsewhere in the genome, but the donor element (blue box) still exists at the original site. Although class II TEs (DNA transposons) also act via transcription and translation of the enzymatic machinery, the integration into elsewhere in the genome results from the excision of the donor element (magenta box).

Sequences of mobile elements, namely transposable elements (TEs), account for an astonishing portion of the host genome in virtually all organisms. In humans, approximately half of the genome is composed of TEs, whereas 1–2% of the genome is composed of protein-coding sequences [1]. Broadly, TEs can be classified into class I (retrotransposons) and class II (DNA transposons), depending on the retrotranscription of an RNA intermediate during their replication cycle (Figure 1). Class I retrotransposons are further categorized into non-long-terminal-repeat (non-LTR) retrotransposons, including long interspersed nuclear elements (LINEs), short interspersed nuclear elements (SINEs), and LTR retrotransposons, which are often referred to as endogenous retroviruses (ERVs) [2]. Retrotransposons require a step of the reverse transcription and subsequently integrate into a new location in the genome, despite structural differences between LTR and non-LTR retrotransposons. Class I retrotransposons, also known as “copy-and-paste” retrotransposons, ideally maintain their original sequence after being integrated into the genome. In contrast, class II DNA transposons mobilize themselves via a “cut-and-paste” mechanism, through which the class II transposase enzyme recognizes its specific terminal inverted repeats (TIRs) flanking its donor transposon sequence and excises both strands at each end. Over time, both classes of TEs accumulate mutations and truncations in their internal sequences, rendering the majority of them unable to transpose autonomously. However, even nonautonomous TEs such as *Alu* and SVA elements can be mobilized in trans by hijacking the L1 enzymatic machinery in new genomic sites [3,4].

**Figure 2:**
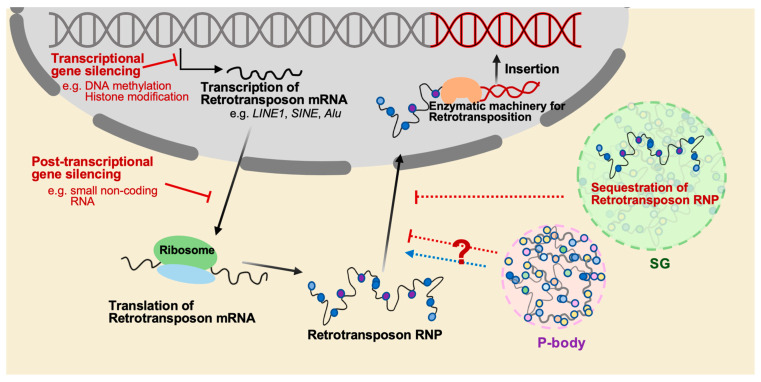
The mechanisms that regulate the mobilization of retrotransposition. The mobilization of retrotransposons is a significant threat to genome stability, and several mechanisms have evolved to suppress retrotransposition. One of these mechanisms is epigenetic regulation such as DNA methylation and histone modification, which can silence retrotransposons at the transcriptional level. Another mechanism is to utilize small noncoding RNAs such as piRNAs in germ cells, which mostly target transposable elements and silence their expression at the post-transcriptional level. Additionally, cytoplasmic granules such as stress granule (SG) and P-body play roles in RNA regulation, including the degradation and storage of transcripts, and may participate in inhibiting retrotransposition through sequestration of retrotransposon RNPs. However, it has also been reported that P-body promotes the mobility of the yeast retrotransposon *Ty*.

Once considered as junk DNA or selfish elements that reside in the host genome, an enormous effort, together with the advent of technical advances, has allowed us to appreciate the impact of TEs on the host genome, not only in terms of local mutations and genome size, but also in terms of deleterious and adaptive outcomes for individuals [5]. Even TE sequences that are no longer mobile can serve as genomic sites for uneven crossing-over, leading to changes in the genome structure [6,7]. TE segments that contain transcription modules, such as enhancers, insulators, and repressors, can contribute to influencing the genome and function of nearby genes by interacting with the host’s transcriptional machinery [8,9]. Numerous cases in plants, insects, and mammals indicate that TEs participate in genomic and phenotypic variability [10,11]. However, their ability to propagate within host genomes can also cause genomic instability and disrupt cellular function with catastrophic consequences [12,13], suggesting that TEs, as genetic parasites, can be a source of genetic conflict residing in the host genome. The consequences of TE activation can be neutral, cooperative, or detrimental, occurring regardless of host fitness. As a result, hosts have developed a variety of defense systems at both the transcriptional and post-transcriptional levels against this activation (Figure 2).

One of the well-known mechanisms to repress TEs is to utilize small noncoding RNAs that are grouped by their size and association with the type of Argonaute proteins. In particular, the PIWI-clade Argonaute proteins predominantly present in the germline associate with a class of small RNAs known as PIWI-interacting RNAs (piRNAs). Initially discovered in the germline of *Drosophila melanogaster*, other organisms, including humans, have employed this type of small noncoding RNA to silence TEs as well [14,15,16,17,18,19]. piRNAs are produced as long precursor transcripts derived from specialized loci in the genome, called piRNA clusters [20]. Exported to the cytoplasm, piRNA precursors are further processed in a cytoplasmic granule called nuage where the PIWI-clade Argonaute proteins associate with mature piRNAs to target TEs [21]. The RNP complex recognizes TEs through the sequence complementarity informed by piRNAs in the cytoplasm or nucleus and executes a series of biochemical events that eventually silence the “non-self” elements via post-transcriptional or transcriptional mechanisms [21]. In addition, transcription of TEs is suppressed through epigenetic mechanisms early in mammalian development and maintained during somatic differentiation [22,23,24]. A large family of Krüppel-associated box (KRAB) domain-containing zinc finger proteins (KZFPs) binds to DNA via zinc fingers and recruits KRAB-associated protein 1/tripartite motif protein 28 (KAP1/TRIM28). This complex recruits several factors such as DNA methyltransferase (DNMT), histone deacetylase (HDAC), heterochromatin protein 1 (HP1), nucleosome remodeling deacetylase complex (NuRD), and SET domain bifurcated 1 (SETDB1) to repress TEs via locus-specific heterochromatin formation [25,26,27,28]. As the tethering of KRAB to DNA promotes DNA methylation during early embryogenesis [29], ZFP57/KAP1 recognizes the methylated TGCCGC hexanucleotide as the motif of imprinting control regions (ICRs) [30]. Especially in TE sequences, the rapidly evolving KRAB zinc finger proteins (KZNFs) associate with different TE families such as ERVs and LINEs. Several studies have supported the idea that KAP1 and Zfp809 target murine leukemia viruses (MuLVs) in mouse embryonic stem cells [31,32]. Approximately three-fourths of KAP1 associated within ERVs is required for the establishment of chromatin-repressive marks by SETDB1 [33]. Epitope-tagged KRAB-ZFPs in human and mouse cell lines show their preferential binding to specific families of retrotransposons [34,35,36]. Furthermore, depletion of KAP1 or the histone methyltransferase SETDB1 in murine or human ESCs can lead to the activation of multiple TEs [37,38].

Recent studies have shed light on new aspects of RNP granules involved in TE regulation that could serve as an additional layer of the host defense mechanisms beyond transcriptional and post-transcriptional silencing of TEs. RNP granules are non-membrane-bound structures consisting of protein and RNA components, which are believed to serve as centers for the regulation of RNA metabolism, including mRNA storage, transport, translation, and degradation. TEs have been found to interact with RNP granules, suggesting that these granules may modulate TE activity through various mechanisms such as TE mRNA sequestration, translational control, and degradation. Therefore, this review aims to discuss the newly discovered regulatory mechanisms associated with RNP granules and their potential implications in the context of TE regulation.

## 2. Cytoplasmic RNP Granules

The control of gene expression in eukaryotic cells takes place at the transcriptional to translational levels. As translation occurs in eukaryotes, mature mRNAs must be exported from the nucleus to encounter the translation machinery present in the cytoplasm. In other words, the localization of mRNAs to subcellular compartments can influence their interaction with various protein complexes, thereby providing a mechanism for the regulation of gene expression at the post-transcriptional level. Cytoplasmic RNP granules are membraneless subcellular compartments in the cytoplasm of eukaryotic cells, which serve as important sites for RNA regulation such as RNA storage and decay [39,40]. Although they consist of various proteins and RNA molecules, their composition significantly differs depending on the cellular context and condition [40]. There are two major types of cytoplasmic RNP granules in somatic cells, namely stress granule (SG) and processing body (P-body) [40].

### 2.1. Stress Granule (SG)

During physiological stress, cells employ various defense mechanisms to counteract the continuous threats to their functionality and survival. SG is a prominent cytoplasmic RNP granule that plays an important role in coping with physiological stress [41]. Once cells are under certain types of stress such as heat shock, oxidative stress, viral infection, and nutrient starvation, SG forms in the cytoplasm to adapt to and overcome stress stimuli by regulating cell signaling and gene expression, which eventually affects cell fate, such as cell growth, apoptosis, and senescence [40,41,42,43,44].

Although various stress stimuli can induce SG, the assembly of SG is typically associated with translational suppression [45]. SG targets and stores mRNAs, ribosomal subunits, translation initiation factors, and RNA-binding proteins (RBPs) [46]. Cellular stress can trigger the phosphorylation of eIF2α, which is commonly thought to be responsible for inducing SG formation [43,47]. However, SG formation can also be achieved by dissociation of the eIF4F complex regardless of eIF2α phosphorylation [48]. These pathways allow for the release of mRNAs from polysomes, leading to SG assembly and translation inhibition [46,48].

Traditionally, the primary function of SG was thought to inhibit mRNA translation. However, emerging evidence increasingly challenges this understanding, suggesting that SG may not necessarily interfere with mRNA translation. Recent research using single-molecule imaging of translating RNAs has shown that many RNA molecules undergo translation in SG [49]. Additionally, earlier research using RNA-seq analysis demonstrated that only a small portion of translationally suppressed mRNA transcripts are incorporated into SG during stress [50]. These observations raise several compelling hypotheses. Firstly, SG may harbor a mechanism for selective translation. Secondly, the role of SG could be conditional, varying based on the type of stress or the specific cell type. Finally, distinct types of SG may exist within the same cell, each performing different roles in mRNA translation. For instance, some SGs primarily silence mRNAs, but other SGs facilitate selective translation. These hypotheses provide a more intricate perspective of SG in stress response and mRNA translation. Further research into these intriguing possibilities could reveal more detailed stress response mechanisms, potentially enhancing our understanding of cellular physiology.

RBPs containing intrinsically disordered regions (IDRs), such as PABP1, TIA1, and G3BP1, play an essential role in SG formation [46,51]. IDRs are protein segments that are distinguished by their significant flexibility and diverse structural conformations [52]. The capability of RBPs to bind RNAs, along with the flexibility of their IDRs, means that they effectively act as nucleators for SG formation [46,51,52]. These RBPs, with their unique physical and chemical properties, undergo a process known as liquid–liquid phase separation (LLPS) to initiate the assembly of SG [53,54]. During LLPS, the proteins undergo a transition from a homogeneous mixture to two distinct phases. One phase becomes enriched with these specific proteins, while the other phase maintains the characteristics of the initial cellular environment [52]. The enriched phase serves as the core structure of SG, establishing a concentrated microenvironment where biomolecules are sequestered [52,53,54]. As SG matures, additional proteins and transcripts are incorporated into the shell layers through protein–protein, protein–RNA, and RNA–RNA interactions [53,54]. Therefore, regulatory factors that affect these interactions modulate the components of SG and thereby impact their functions. For instance, post-translational modifications (PTMs) of RBPs such as phosphorylation, methylation, ubiquitination, and sumoylation have recently been reported as crucial mechanisms for both LLPS and RNP granule dynamics [55,56,57,58]. These modifications either weaken or strengthen the interactions between macromolecules that undergo phase separation, and selectively attract or repel certain macromolecules into or out of these condensed structures.

#### 2.1.1. Sequestration of RNAs into SG

Recent studies suggest that stress-induced RNA granulation is not solely directed by the translational arrest of bulk mRNA species [49,50]. RNA physical properties, RNA modifications, and RBPs are among the additional factors that can contribute to the sequestration of RNAs into SG. A previous study using transcriptome analyses showed that RNAs found in SG have distinct features including the RNA length and the presence of specific RNA motifs such as AU-rich elements [50]. In addition, RNA modifications, such as N6-methyladenine (m6A), can affect the sequestration of transcripts into SG [59,60]. Poly-methylated mRNAs with multiple m6A residues are more highly enriched in SG than nonmethylated or monomethylated mRNAs [59,60]. In addition, the selective binding of RNA-binding proteins such as ZBP1 and Rbfox2 to specific target mRNAs within SG can lead to their stabilization [61,62]. Once the stress conditions have been removed, this stabilization may contribute to increasing the rate of protein production, highlighting the potential role of SG in regulating mRNA stability and translation inhibition during stress. Conversely, it is also presumed that RNAs targeted to SG may undergo degradation because SG and P-body can physically interact and fuse together under certain stress conditions [40]. This is supported by the fact that P-body is thought to function in RNA decay through the presence of mRNA decapping enzymes and exonucleases such as Dcp1/2 and Xrn1 [40]. In line with this idea, single-molecule imaging has shown the movement of mRNA molecules between SG and P-body, although this process occurs at a relatively low frequency [63]. Overall, the findings suggest that SG plays a crucial role in regulating mRNA stability and translation inhibition during stress by sequestering specific RNAs based on their physical properties, modifications, and interactions with RNA-binding proteins.

#### 2.1.2. Cellular Signaling and Apoptosis

SG actively participates in a variety of cellular processes such as metabolism, cellular signaling, apoptosis, and cellular senescence. SG harbors signaling proteins to modulate their function. For instance, the RACK1 protein that activates stress-responsive MTK1 is located in SG under severe stress, leading to the suppression of apoptosis [64]. SG also prevents mTORC1-hyperactivation-induced apoptosis by sequestering RAPTOR to inhibit its association with mTORC1 [65]. In addition, independent studies showed that DYRK3 and Hsp90 contribute to mTORC1 regulation by controlling SG disassembly [66,67]. Under stress, DYRK3 dissociates from Hsp90 and relocalizes to SG, leading to mTORC1 inhibition by facilitating SG assembly [66]. After stress relief, DYRK3 re-establishes its interaction with Hsp90 to stabilize and activate itself, which, in turn, encourages SG disassembly to restore mTORC1 signaling [67]. This feedback mechanism may help balance SG during stress and impact cell survival. Meanwhile, SG formation is associated with cellular senescence. SG can attenuate cellular senescence by sequestering PAI-1 and decreasing its secretion, which promotes cell cycle progression and upregulates nuclear cyclin D1 [68]. These studies demonstrate that SG modulates signaling pathways and influences cell fate decisions by changing the localization of kinases and other signaling proteins.

#### 2.1.3. Antiviral Function

During viral infection, host cells display a dynamic relationship with SG [69]. SG formation occurs as a part of the integrated stress response (ISR), which limits viral replication by activating host innate immunity signaling pathways [69,70]. Protein kinase R (PKR), a key component in the antiviral innate immune system, becomes active upon binding to double-stranded RNA (dsRNA) in viruses with dsRNA genomes, hairpin structures in single-stranded RNA (ssRNA) viruses, or viral replication intermediates [71]. Once activated, PKR triggers the integrated stress response (ISR) to inhibit global translation, resulting in the formation of SG [71,72]. SG also serves as a platform for the activation of innate immune signaling pathways and promotes the production of interferons and other cytokines [72]. During G3BP1-induced SG formation, stress-responsive kinases such as JNK activate transcription factors including NF-κB and IRF3. This activation leads to the production of pro-inflammatory cytokines and type I interferons (IFNs) [73]. Type I IFNs activate the JAK-STAT signaling pathway and induce the expression of IFN-stimulated genes (ISGs), which directly inhibit viral replication and promote the clearance of the virus [74]. Moreover, SG also recruits and activates proteins involved in RNA sensing and signaling, such as RIG-I and MDA5, which detect viral RNAs and activate the production of type I IFNs [72,75,76]. RNA sensing and signaling pathways promote the activation of IRF3 and the production of pro-inflammatory cytokines, leading to the recruitment of immune cells to the site of infection and the clearance of the virus [74].

On the other hand, some viruses have evolved mechanisms to either inhibit or utilize SG to avoid antiviral activity and evade their replication. For instance, viruses can inhibit SG formation by sequestering or modifying SG components such as G3BP1, which has been shown to facilitate the replication of many viruses, including the hepatitis C virus (HCV) [77,78]. The HCV polymerase NS5B directly recruits G3BP1 to sites of viral replication to enhance viral RNA replication and assembly [77,78]. Similarly, the SARS-CoV-2 virus, which causes COVID-19, has been shown to target G3BP1 to disrupt SG formation and evade host immune surveillance [79,80]. The interplay between SG and viral infection is a complex and dynamic process, with viruses using various mechanisms to either inhibit or exploit SG to promote their replication and evade host immune surveillance.

### 2.2. Processing Body (P-body)

Processing body (P-body) is a cytoplasmic RNP granule that exists in all eukaryotic cells. The structure is characterized by its heterogeneity and dynamic nature with variable sizes and compositions, depending on the specific cellular conditions. Although present in nonstressed cells, P-body increases in both number and size in response to glucose starvation, osmotic stress, growth to the stationary phase, and DNA replication stress [81]. Like other membraneless RNP granules such as Cajal body, nucleoli, and SG, P-body formation depends on protein–RNA interactions, intrinsic disordered protein regions, and LLPS [82].

#### 2.2.1. Composition of P-body

P-body consists of a variety of RNA and protein components that play important roles in post-transcriptional gene regulation. The molecular composition of P-body has been extensively studied, leading to the identification of numerous key components that are essential for P-body function. The RNA components of P-body include untranslated mRNAs, microRNAs, and long noncoding RNAs, which can modulate gene expression and post-transcriptional gene silencing. P-body also contains a diverse array of proteins involved in regulating RNA degradation [83,84]. The CCR4-NOT complex is one such group of proteins, facilitating deadenylation and cooperating with GW182 to promote miRNA-induced deadenylation [85]. EDC4 acts as a scaffolding protein, coordinating mRNA degradation through interactions with various P-body proteins including DCP1A, DCP2, and LSM [86,87]. DCP1A and DCP2 are key components of the decapping complex, which plays a crucial role in mRNA degradation [86,88]. In addition, XRN1 degrades mRNA from the 5′ to 3′ end, while LSM1 interacts with mRNA decay factors as a component of the LSM complex to contribute to mRNA degradation [86,88]. GW182 is an important P-body protein that functions as a scaffold for the mRNA decay pathway [86,88]. By interacting with Argonaute proteins, GW182 brings together components of the pathway, such as the decapping activator DDX6 and the CCR4-NOT complex, to promote mRNA degradation and translational suppression [89]. P-body also contains RNA-binding proteins such as TTP that facilitate the degradation of mRNAs containing AU-rich elements, and Pumilio proteins that regulate mRNA translation and stability during development [90]. In brief, P-body contains crucial components that participate in post-transcriptional gene regulation by facilitating mRNA degradation and translational suppression. The exact composition of P-body is known to be diverse and varies depending on the cell type and environmental conditions [91,92]. These components may collaborate to perform various functions, but further research is still necessary to fully understand their complex interactions and roles.

#### 2.2.2. Function of P-body

Although extensive studies have revealed that P-body contains molecules involved in RNA metabolism, the precise functions and mechanisms of P-body remain unclear. For instance, it is uncertain whether mRNA decay primarily occurs within P-body or elsewhere in the cytoplasm. While P-body contains various factors involved in RNA degradation, accumulating evidence suggests that P-body may function more as a site for mRNA storage and sequestration rather than degradation, and that mRNA decay can occur efficiently in the absence of P-body [93,94]. However, transcriptomic and proteomic analyses, conducted through P-body purification, have revealed the intricate functions of P-body in regulating mRNA expression beyond decay and storage [83]. One study demonstrated that P-body is a dynamic condensate that recruits specific mRNA regulons to repress their expression, suggesting a potential role in mRNA regulation [83]. In addition, it has been shown that P-body-associated mRNAs have reduced translation and stability, indicating that P-body may also function as a reservoir for repressed mRNAs [83]. In contrast, the involvement of P-body in the DNA damage response has been investigated in yeast. Different genes, distinct from those involved in the canonical glucose deprivation/osmotic stress pathway, regulate P-body formation induced by DNA replication stress [95]. It has been identified that the components Pat1, Lsm1, and Asc1 play a crucial role in the hydroxyurea (HU)-induced P-body assembly pathway [95]. Interestingly, when these components are deleted, the cells become more sensitive to HU [95]. In a different study, P-body was found to play a crucial role in the cellular response to DNA replication stress induced by HU. The study found that the regulation of several mRNA transcripts, including HHT1, ACF4, ARL3, TMA16, RRS1, and YOX1 [81], by P-body is essential for replication stress resistance. The key P-body protein Lsm1 has been identified as the regulator of the abundance of these mRNA transcripts, preventing their toxic accumulation during replication stress [81]. The study also observed that YOX1 mRNA localizes to P-body in live cells, indicating that P-body is directly involved in degrading YOX1 mRNAs [81]. Furthermore, the study proposed a model where P-body formation in response to replication stress reduces the pool of YOX1 mRNAs available for translation, enabling cells to upregulate a program of gene expression necessary for replication stress survival [81]. Overall, the study revealed the extent and targets of P-body regulation during the DNA replication stress response. These findings collectively suggest that P-body may be involved in regulating multiple pathways involved in the DNA damage response, potentially through interactions with both RNA- and DNA-processing mechanisms.

## 3. Current Understanding of TEs Related to Cytoplasmic RNP Granules

### 3.1. Stress Granule (SG) and TE Regulation

Among class I retrotransposons, one of the heavily studied TEs is long interspersed nuclear element 1 (*LINE1* or *L1*). Although *L1* makes up about 17% of the human genome and most *L1* copies in the genome are considered to be incompetent to mobilize autonomously, about 100 *L1* copies are known to propagate in the genome, with the estimation of a new insertion in every 20–200 births [96]. The full length of the active *L1* elements is about 6 kb, containing a bidirectional promoter in a 5′ UTR region, a 40 kDa ORF1 protein, a 150 kDa ORF2 protein, and a 3′ UTR region with a poly(A) tract. The process of *L1* retrotransposition requires both the ORF1 protein, which encodes an RNA-binding protein with nucleic acid chaperone activity, and ORF2 protein, which has endonuclease and reverse transcriptase activities [97].

In addition to the defense mechanism against *L1* mobilization by the host, growing evidence indicates that *L1*s are localized in cytoplasmic RNP granules such as stress granules. Transfected constructs and endogenous human *LINE1* ORF1p, as well as SW1 ORF1p (related non-LTR retrotransposon from teleost fish), are enriched in cytoplasmic foci with a diffuse cytoplasmic expression, which have a similar pattern to cytoplasmic aggregates of endogenous mouse *L1* ORF1p in embryonic carcinoma (EC) cells [98,99]. These foci overlap with several SG markers such as TIA-1, endogenous elongation initiation factor 3 (eIF3), and PABP. Engineered human *L1* constructs containing tagged ORF1/2 as well as *L1* mRNAs are also co-localized with SG markers, further suggesting a role of SG in the life cycle of *L1*s [100,101].

Mounting evidence indicates that localization of *L1*s in SG appears to negatively impact on their retrotransposition events (Table 1). The zinc finger antiviral protein (ZAP), a type I interferon-stimulated gene, plays a role in the specific loss of viral messenger RNAs (mRNAs) from the cytoplasm [102]. ZAP physically interacts and co-localizes with engineered human *L1* RNPs as well as mouse *L1* and zebrafish *L2* in SG [103,104]. Given the function of ZAP in the destabilization of *L1* RNAs and retrotransposition events, SG might be the cellular compartment involved in RNA metabolism to limit the stability of *L1* RNAs and/or inhibit translation. Myxovirus resistance protein B (MxB), another interferon-stimulated gene, also restricts *L1* retrotransposition by enhancing the sequestration of ORF1p to G3BP1-containing cytoplasmic granules [105]. As MxB inhibits the nuclear import of HIV-1 and herpesvirus [106], the sequestration of *L1* RNPs into SG might prevent the accession of *L1* RNPs to the nucleus for their reverse transcription. In addition, SAM domain and HD domain containing protein 1 (SAMHD1), involved in Aicardi–Goutières syndrome (AGS) as an improper immune activation [107], is an antiviral factor that inhibits human immunodeficiency virus type 1 (HIV-1) and herpes simplex virus 1 (HSV-1). Recently, it has been shown that SAMHD1 controls *L1* retrotransposons [108,109]. Besides its enzymatic function in decreasing the dNTP level necessary for DNA synthesis, SAMHD1 can induce the formation of SG and lead to the sequestration of *L1* RNPs into SG, subsequently resulting in the restriction of *L1* retrotransposition. Lastly, the formation of SG induced by hepatitis C virus (HCV) infection shows a reduction in human *L1* retrotransposition in hepatoma cells where *L1* ORF1p is trapped within the SG. Collectively, these reports suggest that the sequestration of *L1* components into SG inversely correlates with retrotransposition [110].

In addition to *LINEs*, *Alu* elements belong to the class of retrotransposons termed as short interspersed elements (SINEs). There are approximately 1 million copies of *Alu* elements in the human genome, which are known to be nonautonomous retrotransposons. Despite the requirement of transacting factors for their propagation, *Alu* elements have successfully continued to mobilize in the genome. They undoubtedly contribute various aspects to their host such as genetic diversity and diseases [111]. Interestingly, SG might participate in controlling *Alu* elements to some extent. The expression of the GFP reporter containing inverted *Alu* repeats at its 3′ UTR was found to be suppressed but prominently localized in cytoplasmic SG [112], indicating that the expression of inverted *Alu*-containing mRNAs can be inhibited in SG. Similarly, even though exogenously expressed *Alu* RNA alone is typically found in the nucleus, it has been demonstrated that when *Alu*-like 7SL RNA containing MS2 stem loops and GFP-tagged *L1* ORF1p are co-expressed, they co-localize in cytoplasmic granules [101].

Comprising approximately 8% of the human genome, a complete human endogenous retrovirus (*HERV*) consists of 5′- and 3′-long terminal repeats (LTRs) flanking a Gag-Pro-Pol protein-coding sequence [113]. The common set of genes includes the Gag gene, which encodes the structural protein of the viral particle; the Pro gene, which encodes the viral protease; and Pol, which produces the enzymes for reverse transcriptase, integrase, and ribonuclease H. Although related to infectious retroviruses, most of them remain either inactive or silenced by truncation/mutation or host defense mechanisms such as methylation. However, intrinsic *HERVK* mRNAs and proteins are expressed for a short time period during development, and cis-acting elements in the *ERV*s can act as an enormous reservoir of gene regulatory modules under various conditions [114,115,116,117]. Similar to *L1* localization in SG, *ERV* also appears to be localized in SG to some extent. Staufen-1 is required for the development of the anteroposterior axis during embryogenesis [118]. It has been shown that Staufen, as an RNA transport protein, is localized in SG [119]. Staufen-1, as a cellular binding partner for the Gag protein of *HERVK* (HML-2), localizes *HERVK* Rec proteins in SG induced by sodium arsenite treatment [120]. Another report showed that when ALS-associated aggregating forms of TAR DNA-binding protein 43 (TDP-43) were overexpressed in astrocytes, *ERVK* proteins aggregated in the cytoplasm, and their localization was within G3BP1-positive SG [121]. Astrocytes in this context are able to clear *ERVK*s via autophagy, suggesting a potential interplay between SG and autophagy.

Moreover, genetic factors related to TE silencing have been linked to SG as a potent site for TE regulation. In various cell culture systems, members of the cytidine deaminases, APOBEC3 family (apolipoprotein B mRNA-editing catalytic polypeptide-like 3, such as hA3A, hA3B, hA3F, and hA3G), have been shown to play an inhibitory role in the retrotransposition activity of *L1* [122] as well as *Alu*, murine *IAP*, and *MusD* TEs [123,124,125,126]. In parallel, several APOBEC3s have also been shown to be present in SG [127,128,129,130]. Although the mode of action of SG on the inhibitory aspect remains to be elucidated, a family of APOBEC3s could restrict retrotransposons via deamination-dependent or -independent mechanisms [129,131,132,133], depending on the context of cell types and experimental conditions. Another example is MOV10 (vertebrate homolog of *Drosophila armi* gene), homologous to MOV10-like-1 (MOV10L1), which interacts with the piRNA pathway proteins MILI and MIWI [134,135]. MOV10 is a putative RNA helicase, first discovered as a protein that blocks the infection of Moloney murine leukemia virus. When overexpressed, MOV10 has been shown to suppress the retrotransposition activity of *L1*s, *Alu*, and *SVA*s in cell culture. This is unlike other RNA helicases that are associated with *L1* ribonucleoprotein particles (RNPs) [136,137]. Due to its localization in SG and association with Argonaute proteins and uridyltransferases [138,139], it is tantalizing to speculate that MOV10 might recruit *L1* RNPs to SG to silence/degrade *L1* via RNA interference (RNAi).

### 3.2. Processing Body (P-body) and TE Regulation

As a type of LTR retrotransposon analogous to retroviral proviruses but lacking an envelope gene, *Ty* elements are extensively studied retrotransposons in the budding yeast *Saccharomyces cerevisiae* [140,141]. Transcribed by RNA pol II, transcripts from *Ty1* and *Ty3* are exported to the cytoplasm where they assemble into nucleocapsids, known as virus- like particles (VLPs). Genetic evidence has suggested that *Ty1* and *Ty3* require host factors related to P-body to complete their replication during their life cycle (Table 1). For instance, several studies using mutants in yeast have shown that host factors, such as the decaysome Lsm1p-7p complex, Pat1p, Dhh1p, and Pop2p, are required for the retrotransposition of *Ty1* and *Ty3* [142,143,144]. Consistent with these observations, fluorescent protein-tagged *Ty1* and *Ty3* have been found to associate with P-body proteins, and their mobility has been shown to be impaired in P-body mutants such as dhh1Δ and kem1Δ strains [145,146] as well as 5′ and 3′ mRNA decay pathway mutants [147,148], supporting the hypothesis that P-body serve as subcellular regions for promoting the assembly and mobility of *Ty* VLPs.

Comprising ~12% of the mouse genome, intracisternal A-type particles (*IAP*s) are endogenous retroviruses that actively mobilize in the mouse genome [149,150,151]. In contrast to the supportive role of P-body in the replicative cycle of the retrovirus-like *Ty3* element in yeast [145], it has been suggested that P-body participates in suppression of the retrotranposition of *IAP*s [152]. The knockdown of factors necessary for the formation of P-body in human cells such as RCK/p54 or eIF4E-T increases the steady state of *IAP* mRNAs and the Gag protein and causes a shift in *IAP* mRNA from a nonpolysomal to a polysomal fraction, suggesting that P-body plays a role in inhibiting the retrotranposition of *IAP*s. Additionally, the same group also reported that siRNA treatment against MOV10, a component of P-body, inhibits the retrotranposition of *IAP*s [153].

The electron-dense subcellular structure in the vicinity of mitochondria, named intramitochondrial cement (IMC) or chromatoid body from male germ cells [154,155], has been suggested as a location for RNA storage and processing [155]. Small discrete bodies with an irregular shape in germ cells often contain a variety of factors related to RNA processing such as GW182, DCP1a, DDX6/p54, and XRN1, which are components of P-body [156]. Interestingly, germ granules with a fraction of the piRNA pathway factors such as MIWI2/TDRD9/MAEL significantly overlap with granules with components of P-body in the fetal male germline, named piP body [157]. The absence of other factors for the piRNA pathway, such as MILI, in piP body indicates the interplay between mRNA degradation/translational repression and the piRNA pathway within distinct cytoplasmic compartments for the critical developmental window of DNA methylation to suppress TEs in the fetal germline. Similarly, in the female fruit fly germline, components of P-body such as decapping protein 1/2 (DCP1/2), TRAL/Me31B (Trailer hitch/Maternal expression at 31B), and pacman (PCM) partially overlap in cytoplasmic foci with piRNA pathway components such as Aub, Ago3, and Krimp [158]. It has been shown that transgenes from the 3′ UTR of the *Het-A* or the *I-element* retroelement fused to the bacteriophage MS2 overlap in PCM, and that the level of the *Het-A* transcript is derepressed in dcp1 and tral/me31B mutants, suggesting the role of cytoplasmic granules in removing full-length transcripts and/or decay [158,159].

**Table 1:** Studies showing localization of TEs in RNP granules.

Type of RNP Granule	TE	TE Component	Co-Localized with RNP Granule Components	References	Effect on Retrotransposition
Stress granule	*LINE1*	ORF1p (engineered) ORF2p (engineered) ORF1p (Antibody) ORF2p (Antibody) 3′ UTR (MS2 fusion) *L1* 5′ UTR of LINE1	TIA1, G3BP, HuR, eIF3η G3BP, eIF3η TIA-1, G3BP, eIF3η Co-localized with ORF1p TIA-1 Co-localized with ORF1p	[99,100,101,104,105,109,129] [100] [99,101,103,104,105,109,110] [101] [100,101,103,105] [104]	Inhibitory: [103,104,105,109,110,152] Supportive: [146,147]
	*SINE1 (Alu)*	*Alu*-like 7SL RNA (MS2 fusion) Transgene with inverted *Alu* repeat	Co-expressed with ORF1p eIF3η	[101] [112]	
	*ERV*	*HERVK*(HML-2) Reverse transcriptase	TIA-1 G3BP1	[120] [121]	
P-body	*Ty*	*Ty1* GAG *Ty3* POL3/GAG *Ty1* mRNA	Dhh1, Dcp2 Xrn1, Dhh1, Pat1, Dcp2, Ded1, Lsm1	[146,147] [145,148] [146]	
	*IAP*	*IAP* gag mRNA	RCK	[152,153]	
	*Het-A* *I-element*	*Het-A* and *I-element* (MS2 fusion)	PCM	[158]	
	*Alu*	Transgene containing inverted *Alu* repeat at 3′ UTR	GW182	[112]	

## 4. Concluding Remarks and Future Perspectives

Recent studies have suggested that RNP granules such as SG and P-body may be potential candidates for TE silencing. Although the localization of transposable elements (TEs) does not necessarily imply their suppression, growing evidence indicates that the localization of TE ribonucleoproteins (RNPs) in SG can reduce the retrotransposition rate. Furthermore, certain factors within SG and P-body have been shown to suppress TEs. This evidence collectively suggests that RNP granules may regulate TEs, in addition to their role in translational attenuation. However, numerous questions remain regarding the mechanisms of TE regulation within RNP granules. For instance, it is still unclear which core factor(s) is (are) responsible for recruiting TE RNPs into SG, and what the consequences of TE RNPs within SG are. Further research is required to fully elucidate the interaction between RNP granules and TEs during stress and the potential regulatory mechanisms involved.

## Data Availability

Not applicable.
